# Animals in Animal-Assisted Services: Are They Volunteers or Professionals?

**DOI:** 10.3390/ani12192564

**Published:** 2022-09-26

**Authors:** Brigitte Wijnen, Pim Martens

**Affiliations:** University College Venlo, Maastricht University, Nassaustraat 36, 5911 BV Venlo, The Netherlands

**Keywords:** animal-assisted services, animal-assisted interventions, animal-assisted activities, animal volunteer, animal professional, animal wellbeing, animal motivation, context-ethogram, dopamine, wearable EEG, reciprocity

## Abstract

**Simple Summary:**

Animal welfare is a growing concern in Animal-Assisted Services. Although studies have been conducted on stress signals and—to a lesser extent—positive emotions, no research has yet been conducted on the motivation of the integrated animal, to the best of our knowledge. Not all therapy animals are trained to assist. Are they volunteers or professionals? Volunteers have a higher degree of self-government and can quit when they are not motivated anymore. Professionals might, however, go the extra mile. Can we compare animal volunteers and professionals to their human equivalents? If so, this might help to structure discussions about animal welfare and motivation in interventions. Furthermore, it can provide better arguments for the career planning and career ending of these animals. Using animal-friendly interventions might influence motivation and, consequently, the moment at which efforts cease. Studying motivation is not easy, as it requires data on dopamine, the molecule involved in motivation, reward and repetition of behavior. However, the use of wearable techniques such as on site electroencephalograms (EEGs) for freely moving animals and non-invasive dopamine measurements is a developing and promising area of research. The translation of these data into context-ethograms—ethograms that show behavior in a context/intervention—can help handlers and therapists to understand the behavior of their therapy animal better and with less subjectivity.

**Abstract:**

With the increasingly common practice of Animal-Assisted Services (AAS), whether in therapy, coaching, education, or volunteering programs, the concern over animal welfare has also risen. However, no standards have yet been established for good practices to ensure the animal’s mental health. This is largely due to the wide variety of roles played by animals in interventions and the lack of ‘job descriptions’ for the animal in diverse settings. Some professionals call their animal a ‘volunteer’, others mention that some directive guidance is given to the therapy animal, and some assistance animals are highly trained. Misunderstandings could be avoided if the integrated animal were to receive a justifiable label: volunteer or professional. Choosing either one comes with obligations for the owner, handler, or therapist. In this paper, we compare the roles of human volunteers and professionals to the roles of animals involved in therapy. We also demonstrate the obligations that come along with the decision to label animals as such either volunteers or professionals. Furthermore, we make a plea for animal-friendly interventions, whether in a volunteer position or as a professional, in order to stimulate the animal’s cooperation and motivation. Studying dopamine and translating the findings into context-ethograms can provide a way to judge behavior more objectively.

## 1. Introduction

In the past fifty years, Animal-Assisted Services (AAS), previously known as Animal-Assisted interventions (AAI), and Human Animal Interactions (HAI), have largely evolved. At first, this started with merely the presence of animals in therapy sessions, planned or by coincidence, which seemed to have an impact on the client involved [1]. The interventions evolved and the role of the animals became more active and dominant [2], which has recently led to growing concerns relating to the welfare of the animals involved in these interventions [3]. The need for protocols to ensure the safety and wellbeing of both species has emerged, such as quality measurements and best practices [4]. This raises the following question: ‘do animals benefit too?’ [5].

In this discussion paper, we focus on the role of the animal and whether its integration in an intervention should be regarded as voluntary work or a profession. Choosing either one of these options might influence the way therapists and handlers integrate the animal in therapy sessions on the one hand and the competencies of the animal on the other.

We challenge the therapy animal handlers or therapists working with animals to reconsider their animal’s working conditions. Is the animal working as a volunteer or as a professional, and how do we decide the difference? A comparison with the rights of human volunteers and human professionals could help to support and structure the discussion around animal welfare in AAS. Although research has been carried out to study stress in animals, little supportive research has been conducted on their positive emotions during interventions [6], and no research has been conducted on motivation. Motivation can be seen as the driving force encouraging an animal to show and repeat behavior, seeking the reward it anticipates and the salience of the reward—a process modulated by dopamine [7]. In this article, we also suggest a neurological approach in order to obtain a better understanding of motivation driven by dopamine during AAS, and as such obtain a better understanding of the welfare and wellbeing of the animal.

Guidelines for housing, feeding, moving space, communications with co-species, and other physical and mental care are not discussed in this paper, as these have already been covered by the white paper of the International Association of Human Animal Interactions (IAHAIO) [8] and Animal Welfare Indicators (AWIN) protocols [6]. The latter also explains the presence and absence of stress signs in handled animals in HAI; however, in this paper we stress the need to label the animal’s performance first before stating matching welfare conditions. They might be different, and the animal’s motivation could play a central role in this discussion.

The idea of this paper is to encourage and structure the discussion about animal welfare in AAS by using a human model for comparison and taking a closer look at motivation of animals.

## 2. Reconsidering Reciprocal Altruism

Many researchers have theorized about the working mechanisms in AAS, and several models and theories have been proposed across the literature [9,10,11,12,13,14]—few to none of them are supported by robust evidence-based findings in large samples. Another mechanism which has almost been forgotten is the theory of reciprocal altruism. Why do animals cooperate with us?

Although this hypothesis is by no means new in evolutionary biology, the relationship of two species that mutually benefit each other resembles the relationships seen between humans and animals in human–animal relationships and in therapeutic sessions. Reciprocal altruism was first used by the biologist Robert Trivers to describe the behavior in which one organism acts in a way that temporarily reduces its fitness while increasing the fitness of another, with the expectation that the other organism will act in the same way at a later date [15]. Examples are the cleaner wrasse *Labroides dimidiatus,* which swim with the client fish at the risk of being eaten while they clean the skin or the mouth of the client [16], and oxpeckers, which remove parasites from the skin of rhinos by picking them up sitting on their backs and at the same time alarming the rhinos to allow them to detect and evade human hunters [17].

There is a time tension in this hypothesis: the expectation of the bidding organism is that the favor will be returned later. This can be in the form of the food, shelter or attention that the other in the dyad offers. In the end, the equilibrium is evolutionarily beneficial for the fitness of both [18]. In the world of animal-assisted care, we can find similarities in interventions in which animals present themselves to be integrated into the intervention without resistance, sometimes even with visible pleasure and reciprocity. There is also criticism of the theory behind reciprocal altruism, particularly because animals live more in the moment and do not consciously look forward to the future, like humans [19]. Animals would not be cognitively concerned with the investment they now offer and would adopt an expectant attitude towards a possible return. However, Schino and Aureli mention that reciprocity is (not) always cognitively demanding but rather can be seen as emotion-based mental accounting [20]. In practice, it can be observed that the animals in AAS offer a more or less conscious investment—they do not refuse, but cooperate, sometimes with visible pleasure, sometimes stoically [21]. Perhaps the reciprocity is so close that a habituation or conditioning occurs. After work, the animals eat and rest and there is room for social behavior with group members. The efficacy of animal-assisted therapy (AAT) depends heavily on the reciprocity of animals, patients and team members [22].

Altruism in humans and animals feeds the dopamine reward systems in the brain [23]; it feels good to give voluntarily to others, not only to one’s kin [20]. This presumes a kind of interspecies communication, similar to the study of cow language towards the human handler [24].

In AAS, the behavior of trained dogs might disguise their original altruism. It is known that a DNA change in the domesticated dog related to the Williams Beuren Syndrome (also in humans) is the reason for the hypersocial behavior of many dogs. This has been attributed to the rapid divergence of dogs and wolves and is thought to have facilitated their coexistence with humans [25]. However, many untrained animals also show altruistic behavior. Donkeys are an example of animals showing human’s friendly, cooperative behavior, without being trained for it [26]. In AAS, they are merely educated but not trained. Is there an evolutionary benefit for their altruistic comportment?

## 3. Animal Welfare in AAS

In the scientific literature, many studies can be found on the positive effects of AAS on humans of different ages and different diagnoses [27,28,29,30,31]. What we know less about is what the positive experiences are for the animals. It is difficult to read enthusiasm from certain animals. A dolphin’s smile is not a smile, but rather an anatomical formation of its face. Donkeys and dogs are often pleasing animals and do not resist when they are incorporated; in fact, when a dog shows fear signals, a line may have already been crossed. There is a growing body of studies on stress signals in dogs and in horses [32] and on emotions in animals [33]. Glenk pointed out in 2017 the welfare threats therapy dogs encounter during interventions as possible indicators of the animal’s work-related stress [6]. However, reducing stress is a form of negative reinforcement, while positive reinforcement might be more rewarding for animals and contribute to their feelings of wellbeing [34]. Researchers should be cautious, because sometimes it is difficult to measure whether physiological responses such as fluctuations in cortisol come from negative stress or positive excitement [35].

Gentle human–animal interactions can induce positive emotions in cattle and enhance their welfare [36]. Additionally, some, but not many, animals act spontaneously to help humans, trusting in their ability to read human’s gestures [37]. In horses, the Equine Personality Questionnaire (EPQ) has been proven to be valid and useful, except for the part relating to gregariousness in the questionnaire [38]. In short, many animals are quickly satisfied; some help spontaneously, while others have a helping character. However, assessing positive emotions and motivation in animals and their willingness to repeat behavior remains difficult.

One conclusion in an exploratory study is that cats’ and dogs’ emotions are difficult to determine, and that a complete image can only result from studying their full body and body movements in a certain context [5]. This supports the need for field studies when animals are ‘in action’ and leads us to the following dilemma. What do animals do when they are in action and how do they experience that? It is difficult to determine to what extent the question of ‘how do horses feel’ (and other animals, sic) can be answered [39].

The first question that arises is ‘what role do animals play in AAS?’ Are they volunteers who can refuse a task, or should they be considered as professional employees, especially employed for care tasks? However, whatever role the animal fulfills, it is especially pleasant for all parties if the animal is motivated and enjoys its task. This not only concerns welfare factors such as freedom from hunger and thirst, discomfort, pain and fear, and room for normal behavior (Farm Animal Welfare Committee 2009), but also joy, excitement, eagerness to perform, and enthusiasm, including the right to flourish. Social, physiologic benefits and behavioral benefits also count [4]. In the future this might become part of the civil rights of animals, the right on freedom, family reunion, liberty and equality and freedom of choices like the American nonhumanrights.org is striving for.

In the following paragraphs we draw a parallel between volunteering and professional work for humans and animals, to raise questions and stimulate discussions about the role animals play in AAS, which may lead to job descriptions and formal working conditions. Furthermore, we press the need for positive interventions during working life of the therapy animal and ask for awareness of the consequences this will have for the retired animal. Finally, scientific research into the motivation of working animals, which might be reflected by dopamine levels, is needed to assess whether animals are motivated to assist in AAS.

## 4. Volunteers

### 4.1. Animal Volunteers Compared to Human Volunteers

Animals are often called volunteers in AAS, suggesting that they work with pleasure and voluntarily join an intervention. Is that comparable to human volunteering? According to the International Labour Organization (ILO) the definition for volunteering by people is work that someone does unpaid and non-compulsory, for others or for society for at least one hour in a four week or one month reference period. The labor is not profit driven, and does not replace professionals [40].

That might be an excellent label for animals in health care. They provide a contribution without being monetarily paid for but cannot be obliged to do so. The question of how to assess animals’ feelings about having or not having obligations arises. The search for examples in scientific research for “animal volunteers in AAS” did not show one result, contrary to human volunteers working with animals in AAS/AAI. Nevertheless, many handlers active in AAS state their animals always work voluntarily, as a synonym for ‘working with pleasure’. However, therapy animals have not agreed to work based on informed consent in contrast to the humans involved [21]. They need to deal with the client they did not choose. Some animals are incorporated in AAS without any training or selection criteria, but just for the reason being there, like shelter animals [41].

Many domesticated animals naturally have a curious nature and respond positively to attention. That does not mean that they want to participate in all activities, which each animal demonstrates in a different way or not at all. Unlike stress, motivation demonstrated by body language is not always clear. Moreover, many animal owners and experts appear to have an inconsistent way of assessing their animal’s behavior, they often disagree [42].

There are some questions to be answered before stating that the animal involved in AAS is working on a voluntary basis. When considering the animal as a volunteer, are we sure that the animals have chosen to be a volunteer? An animal that turns away from a situation can have various reasons for walking away. Not interested, no added value (sweets), motivation to graze, too many people et cetera. If we assume that he—as a volunteer—can always walk away, we might exclude him involuntarily from the situation. Some animals can see this exclusion as punishment or negative reinforcement.

If we have determined that the animal is voluntarily present in the setting, do we expect anything from him? Or is his presence enough and he decides whether he stays or leaves the context and thus ends the session when leaving? Do we accept this as is where is or do we try to influence his behavior?

Human volunteering is unpaid, non-compulsory, but not without obligation. Agreements are made, which the parties must adhere to. This applies to working hours, remuneration, support, group activities with other volunteers, training cetera. On the other hand, the volunteer work can be terminated by the volunteer at any time. Does that also apply to the animals? Additionally, how can we be sure that the animal no longer wants to be involved? Studies on motivation are needed to judge the animal’s willingness to cooperate.

### 4.2. Animal Rights in Voluntary Work

Human volunteers can stop at any time and can easily make that clear with a written or spoken message. Animals do not have these possibilities. If we regard them as volunteers, how can we assess that they do not feel like it (anymore) including the reason for their restraint? Is the work not satisfying enough? Is it too heavy, too long, too often? Handlers need to be able to read the body language well to understand when the—usually service minded by training—animal really does not want to work anymore. Additionally, perhaps even earlier.

Animal emotions are hard to define and can be explained in different ways. They can be studied in terms of mood and affect, behavior or physiological components [33]. Additionally, there might be more to welfare. Additionally, context could be an important influence on animal welfare. Furthermore, interspecific relationships determine the result of interventions, which directly influences the welfare of the animal [22]. In this paper and the following research we study motivation as an indicator of welfare, a not well documented emotion in therapy-assisting animals.

The discussion of animal rights in voluntary work in AAS can become part of the debate about animal rights in general, where legal rights, and the (in)ability of animals to make their own choices are issues to consider for the future [43].

### 4.3. Benefits from Voluntary Work

Do the animals benefit from volunteering? Therapy sessions can be very emotional, but also cheerful and enjoyable. In addition, it is possible, at least in humans, that positive and negative emotions co-occur during stressful periods [44]. It is not difficult for handlers to estimate whether therapy sessions are stressful, but to what extent. Even experienced handlers can be unreliable observers [42]. Stress is not always recognized in body language as opposed to positive emotions that are clearer to observe. Especially in stoic animals, such as donkeys, negative emotions are difficult to score [26]. There is a need for practical solutions to score animal emotions objectively to balance the subjective human observations. This also counts for the professional work that will be discussed later in this paper.

Therapists can try to make therapy sessions more attractive to the animals involved and focus at evoking positive emotions in humans and animals instead of trying to reduce stress. Frans de Waal points out in his book Mama’s last hug: give the animals something positive to do and you will see that they cooperate voluntarily [45]. This can also apply to AAS. Rewarding the animal for the work carried out might influence his motivation and ensure his participation the next day.

Little is yet known about the impact of human emotions on the animal, and if the animal is really conscious of other species’ emotions when crying, shouting, running, or freezing.

### 4.4. Negative Impacts from Voluntary Work

Animals are allowed to refrain from a session, which is potentially damaging to the human client. A booked group session suddenly stops because the animal leaves the context. If the animal is a volunteer, he can. Many handlers in therapies and treatment even see the added value of this: turning away also is a message and the client is invited to find a way to deal with it. However, some animals also walk away when they do not want to be involved anymore or return to their natural behavior. Especially for young human adolescents with low self-esteem this can cause damage; they can see it as a rejection by the loved animal. It takes a highly skilled therapist to reverse this conclusion. Would not it be better for the therapist to motivate the animal and keep it in the process?

Although leaving the context is mostly not possible, in a fenced or closed environment there never is a real escape, it is important for the handler and therapist to come close to the reason for this behavior. Finally, another important question remains: Can an animal refuse a client?

Human volunteers do not retire; they just quit and there are no obligations left for the employer. That could meet sincere protest in the world of AAS; handlers and owners remain responsible for welfare of the animal until death.

## 5. Professionals

### 5.1. Animal Professionals Compared to Human Professionals

In the human world a professional is someone with special skills, which is mainly characterized by craftsmanship, knowledge and strong competencies. This often refers to people who function at a higher level than volunteers. They have years of experience, a lot of education and training and excel in their work. Can we approach animals as an professional employee? What selection procedure do we use, on what arguments can we state that the animal is motivated, can we test its willingness to work? This requires a different approach than to volunteer work, which is not subject to highly specific training and experience requirements. It is not to be forgotten that a certain degree of voluntariness also applies to a professional. He can also quit his job.

If we confront the therapy animal with complex care questions, clients with strong emotions or aggression, how do we protect the animal from this? Horses can smell emotions in humans by odors of fear and happiness [46] and might react correspondingly. Working with people with Alzheimer and dementia has been proven beneficial for patients, but the dogs included were not in very close contact with the patients. Their role was playing with a ball and listening to commands, in a joyful setting and not too long [47].

Since the human therapist remains the director of the process, alone or together with a separate handler, he should monitor and manage the emotional strain that can be put on the animal. His therapeutic background determines the protocols and activities in which the animal is incorporated. In settings with complex care and frequent emotional outbursts an animal with a strong personality is needed, which is stoic and remains calm in all circumstances. Temperament tests are needed to qualify the capability of the animals, like for donkeys which seem ideal therapy animals but also can demonstrate unwanted traits [48]. Still, passing a test, does not necessarily mean the therapy-assisting animal enjoys his task. Like assistance dogs, which are highly trained to support the physically handicapped client, a therapy animal integrated in complex mental health care might also benefit from a training after a thorough selection. For horses, the selection procedure of mounted police horses might be helpful, also a stressful animal job, but even this procedure cannot avoid that human selection of a suitable horse is mostly based on intuition and sometimes proven wrong [49]. Additionally, this study shows, experience makes the difference.

Job descriptions with explicit criteria and competencies might be helpful in the selection process of professional therapy-assisting animals. MacNamara et al. point at the importance of the context when evaluating the appropriateness of an animal for animal assisting therapies and promotes the need for adequate selection procedures. Moreover, prior to assessing aptitude or skills the expected outcomes should be defined, as well as how the role of the involved animal is defined [50]. Job descriptions and hence the job requirements and training demands can differ between professional animals and volunteering animals. Here, we can observe the thin line between obedience and desirable behavior, compared to intrinsic motivation and desirable behavior.

### 5.2. Compensation

The animal employed in this heavier work, the more complex health care, deserves a compensation, a salary or renumeration. What should such compensation consist of? In addition to good housing, sufficient and high-quality food, socializing with conspecifics, the animal is entitled to compensation for the work it provides.

In this realm, the work of Charlotte Blattner and co-authors on animal labor is interesting [51]. They bring up essential questions such as: Do we regard animals as our co-workers and are they entitled to equal rights and compensations? Should we aim at a collective labor agreement for the animals working alongside humans? What should compensation consist of? Can we assess the animals’ consent in labor?

Beyond the need for compensation through positive interventions, such as mindfulness, play therapy or positive reinforcement training exercises, we suggest involving the client in the reimbursement. The therapy-assisting animal may receive a clear message from the client that he has done his job well. Even in case of emotional and aggressive expressions by the human client, a session should be concluded with a reward for the therapy-assisting animal. A reward might be in increasing oxytocin levels (e.g., brushing), or a dopamine stimulating reward (treat or exclusive attention). The client will benefit as well, since it can feel good to do good because it activates the mesolimbic dopamine system [52].

### 5.3. Secondary Benefits

In addition to salary, a good professional also earns training and education, breaks/rest and vacation, a health plan and sick leave with continued payment. For human professionals this is normal in the Western society. An animal active in complex health care should be adequately rewarded to assure its willingness to work, which is not easy. Another question is if reducing social contact with people is the best way for animals to rest. Most therapy-assisting animals love contact with people and become lonely when they no longer see them. In addition, old learned good habits can be forgotten. It can be counterproductive if they receive too many breaks, rest and holidays [53].

### 5.4. The Right or the Duty to Retire?

Many handlers of therapy animals will agree that these animals have a right to rest after a working life. This demands a solid definition of the word ‘rest’ or ‘retirement’. Living with conspecifics in an environment such as a natural habitat, with reduced contact to people is probably not the proper definition for rest after work life. Furthermore, it is difficult to determine the age of retirement, taking into consideration the variation in life expectancy of the various animal species. The intensity of the work can also be decisive; assistance dogs often work 24 h a day, while therapy horses, donkeys and dogs often have a certain work rhythm of commitment and breaks [53].

If the therapist has been successful in integrating animals into interventions which are pleasurable to the animal, the animal may miss those conditions. It has become accustomed to the rhythm, social interaction with people and pleasant interventions [53].

If the therapist wants to continue his activities with a younger animal, the operating costs of the therapist or handler go up. The older, retired animal remains part of the herd or group and deserves care. This care consists not only of food, supplements and medicines, but also of housing and room for exercise. In larger organizations, this can have an impact on the staffing need to take care of the animals. Given the rising popularity of therapy-assisting animals, we need to prepare for aging animals and its consequences.

Therapists and handlers should ask themselves if they are doing the animal a favor with, an inactive, but quiet life or an activity program for seniors. Retirement comes with liabilities.

We also need to consider the retirement of the handler/therapist or another reason to quit work. What happens to the animals? Some animals have a high life expectancy, e.g., a (therapy) donkey can live up to 40 years and some even more.

### 5.5. Do They Enjoy Their Job?

Re-examining the animal/human partnership from the animal’s viewpoint is a prerequisite to imagine what the benefits might be for the animal. Raising, training, and use of therapy and assistance animals is sometimes causing significant degradation in their welfare [3]. A human professional chooses a job he presumably likes and not the job that causes the least stress. Handlers and therapists working with animals need to address the motivation of the animal involved. However, biomarkers for motivation, such as dopamine, have not been studied in dept in the diverse sectors of AAS.

Human jobs can be stressful but very meaningful at the same time. A human oncologist will most certainly have a stressful job but can also enjoy his work. It is—in most cases—a personal choice, and not decided upon by a third party who feels he has the adequate character for it. Let us extend this comparison for a moment. What makes the work attractive to an oncologist can be the variation in his work—that stress is interspersed with fascinating, successful and instructive moments, that his discipline develops, that he has a good relationship with his clients who are grateful for his care and personal interest. If the relationship between therapy-assisting animal, therapist and client is seen as a cooperation, as a collaboration on the way to the best result, in which the animal is also rewarded for his efforts and is integrated in interventions that are not too mentally disturbing, there is a reasonable chance that the animal will enjoy his ‘work’. His job must be safe and without physical strain. To evaluate the animals emotions during its activities we need studies on conscious feelings as one component of emotional states and the neural correlates of consciousness and all the methodological challenges that go with it [54]. For other biologists in studying the behavior of animals, even without neurological data there is already proof of emotions [45].

### 5.6. Dismissal

If an animal is not suitable, there should be a follow-up plan to safeguard it’s future. Does the animal have the right to stay in the group or does it have to leave? Is it succeeded by another animal, forcing the handler to maintain two animals? What facilities are there for the therapy-assisting animal, after retirement of its owner or itself? Can the retired professional therapy animal still join therapy sessions as a volunteer? These questions have not yet been answered thoroughly by the sector but are already highlighted by Ng and Fine in their considerations on retirement of therapy dogs [53].

## 6. A New Challenge: The Non-Invasive Neuroscientific Approach

In the world of AAS, there is little insight into the effectiveness of working mechanisms, although several theories exist. A lot of attention is paid to behavior as a result of suspected stimuli, both in humans and animals [21]. When looking at behavior, many factors play a role. Inspired by the book ‘Behave’ by Robert Sapolsky, we can depict behavior as a result of an evolutionary history [52].

Human behavior is the sum of evolutionary development, from development to the upright human being, the history of thousands of years through the hunter/gatherer living to today’s technical society, the fertilization by which a person receives his genetic package from two parents, the period of pregnancy and birth, the childhood that follows and adolescence where the prefrontal cortex is not yet developed, the memory of events, the hormones that circulate until finally the last step, the neurotransmitters determine what behavior can be seen. Behavior is a sum of history and biology shows that this is also applicable to animals [52].

If we regard neuroscience as an explanatory discipline for animal behavior during interventions with humans, a problem immediately arises; neuroscientific data relating to animal neurotransmission are mainly derived from experiments in laboratories with captive laboratory animals and human subjects linked to neuroimaging devices. Testing neurotransmission is often performed on rodents linked to an electrode. Many of these studies cause long term stress to the animals involved [55], and the animals need special training to enter a functional magnetic resonance imaging (fMRI) machine [56]. Human subjects though, cooperate voluntarily based on informed consent and agree to be linked to an fMRI to monitor brain activity. However, not only is free movement a necessity to reflect the outcome of certain internal cognitive processes in animals, it is also a fascinating field of research since certain crucial behavioral patterns can only be observed and studied during free movement [57].

Funding institutions in the field of AAS often demand non-invasive research methods. If we want to know what humans and animals think and feel during an intervention in the natural environment, real-time neurological data are needed from freely moving human subjects and animals. Heavy equipment can intrude an intervention and muscular activity can cause alteration of, e.g., electroencephalogram (EEG) imaging. Furthermore, it requires anesthesia to implant electrodes in animals which is only possible in clinical settings [58]. Especially when it concerns large animals, such as horses and donkeys, it is difficult to avoid the influence of lab equipment attached to the body on behavior. Animals integrated in AAS are mostly freely moving and not used to the equipment attached to them. Moreover, some interventions, such as paddock exercises, training of dogs by inmates, or open-air mindfulness exercises are not possible at all in lab conditions. However, this also limits robust research results since, unlike in laboratories, the protocols in AAS vary widely [59]. Nevertheless, discussions, studies and research on animal welfare should exclude invasive research as it is opposite to welfare.

In recent years, it has become increasingly possible to perform non-invasive measurements of biomarkers, such as cortisol levels in hair and saliva and oxytocine in urine, which are thought to explain behavior. Cousillas et al. developed a wearable EEG recorder for freely moving horses which was tested on grazing horses in the meadow [58]. With this device Stomp et al. showed that individual EEG profiles in horses are associated with different welfare scores and that horses in a good welfare state produce fewer gamma waves in the right hemisphere [60]. Additionally, d’Ingeo et al. performed a study on brain activity in horses and concluded that associating brain and behavior analysis, clearly demonstrate that horses’ representation of human voices is modulated by the valence of prior horse-human interactions. Horses seem to recognize and interpret human voices [61,62].

The above-mentioned studies were performed on freely moving animals. Studying real-time welfare states in animals during AAS is becoming more and more possible by technical solutions.

While the in vivo detection of neurotransmission within the lab is developing rapidly [63], real-time monitoring of neurotransmission is much more difficult outside the lab. However, it is neurotransmitters that determine the real-time behavior. The impact of hormones is slower and is influenced by brain activity, e.g., the reduction in impulses driven by the amygdala by the prefrontal cortex [64].

In 2000, Odendaal et al. published a study on various neurotransmitters and hormones in blood plasma, a study that nowadays would encounter ethical objections. In their research, oxytocin emerged as the most significant increased hormone, lasting until after the intervention, the hormone associated with social bonding and feelings. Dopamine, ß-endorphin, oxytocin, prolactin, phenylacetic also showed significant increase but only measured close to the intervention moment. According to the authors this study shows that the animals and the humans are experiencing the same physiological effects [65].

Taking this as a premise, a new insight might offer some new information. Neurotransmitters and their breakdown components seem to leave the central nervous system at night, so the moment of measurement might influence findings. Neurotransmitters are broken down in different molecules by enzymes and leave the brain through the glymphatic system, a macroscopic waste clearance system from the central nervous system that functions mainly during sleep [66]. This suggests that the most relevant moment for measuring neurochemicals would than be a day after the intervention, while the hormone cortisol can already be detected up to 10 min (not longer) after an intervention in saliva [67]. Studies are needed for the validation of the theory that neurotransmitters can best be tested the morning after an intervention.

## 7. Stress Reduction Versus Motivation Increase

This overview is by no means a plea to refrain from integrating animals in care. However, it does advocate the introduction of interventions that are as pleasant as possible for the animal, whether it is integrated as a volunteer or as a professional. Pleasure is often key in reward and motivation, for which dopamine is an interesting biomarker. A dog that goes out for a walk often considers this as a great reward, and at the same time it can be part of an intervention: an intervention that does not cause the animal stress. Moreover, the intervention—the walk, regardless of the conversation that the therapist is having—fits in with its natural behavior.

Assistance dogs help with opening doors, using washing machines and picking up mail. They do not perform natural behavior; it is trained. These dogs are often trained in a reward-oriented manner and therefore continue to repeat the desired behavior. If the behavior is rewarded alternately, the dog continues to strive for the correct execution of the wanted behavior and reward. It is the anticipation of the reward that keeps the animal focused. The “virtual” (anticipated) reward is mediated by dopamine [68].

What if the animal is highly motivated and likes to be near people? What if we just skip the volunteer position and only work with animals that clearly want to be involved in therapy? The handler then could manage the animal’s involvement and influence staying or leaving. However, force will induce stress.

Focus on the reduction in stress is not the way; we need to improve motivation. If an animal is often under stress at work, the work is not suitable. However, if we influence motivation with better and more enjoyable working conditions, we can expect something in return.

### 7.1. The Client’s Position

The therapies in which animals are involved are predominantly complementary therapies and not curative treatment methods. Animals are hardly integrated in the treatment of life-threatening diseases but rather incorporated to improve the quality of life of a human client. Although encouraging results have been achieved in people with very low consciousness [69] and people with dementia [70], most interventions are developed for people who are approachable and can perform actions on request. In these circumstances, the therapist and/or the handler could invite the client to enter a collaboration with the animal, working not only on the relaxation of the client but also on that of the animal. Where two parties enjoy an intervention, the result could be positively intertwined. Possibilities are, e.g., mindfulness, relaxing techniques, play therapy, and enjoyable training exercises where both humans and animals experience positive feelings. Therapists could involve clients in animal-friendly games.

The client can also be encouraged to express his appreciation in an animal-fun way, such as a small reward, petting, and complimenting. Dogs, in particular, are good at understanding people’s facial expressions [71], tonal language and body language. More animals also understand these. Elephants can understand the difference between the language of harmless tourists (friendly) and the language of the shepherds they must watch out for (aggressive) and adjust their behavior accordingly [72].

### 7.2. The Position of the Handlers

As indicated earlier, handlers do not fully agree in reading the body language of their animals. This makes it difficult to develop guidelines that translate body language into labels people can understand. A distinction must also be made between stress-induced body language, neutral body language and positive body language or enthusiasm. In certain animal species, this is easier than in others; enthusiastic behavior in dogs is easier for a layman to read than, for example, enthusiasm in alpacas or donkeys.

Organizations such as IAHAO and AWIN provide information about signals that can be interpreted as stress signals or neutral, but no information about enthusiasm or motivation is provided, while motivation is seen as an indicator of pleasure. Motivation is recognizable by the will to repeat the behavior to receive a reward, or to repeat an activity that is experienced as pleasant. Animals can give signals for this [73]. Handlers must be open to reading these signals and work on their recognizability.

Animals respond to stimulation through their senses, such as the production of oxytocin and dopamine when touched [65]. Oxytocin and dopamine are often released together [74]. Handlers can use exercises that have been proven to stimulate oxytocin and dopamine. This means that more research needs to be conducted on the effect of certain interventions on animals. After all, the effect on humans is already being extensively investigated.

### 7.3. The Value of Context-Ethograms

Ethograms often fall short in the interpretation of animal welfare during interventions because they picture behavior and not emotions. Some animals show similar behavior in positive and in negative or sick mood (The Equine Pain and Welfare App). A donkey that stands still with the head down may sleep, be sick or enjoy grooming. It is therefore about the context and the interaction with the human being, and we would therefore like to argue here that more research should be conducted on context-ethograms during interventions based on dopamine findings. The full context is needed to interpret a certain behavior.

A context-ethogram is not the same as an ethogram of behavior in a context. It is the interaction with the human that defines the qualification of behavior in an intervention and not the environment. Does the animal have eye-to-eye contact with the client, is the animal showing behavior to stimulate the client to repeat a behavior, is the animal approaching the client for contact, are the ears of the animal directed to the clients voice? Is the animal moving backwards after clients’ behavior?

Studying context-ethograms during interventions and combining the information with data on dopamine might result in more reliable conclusions for welfare.

## 8. Discussion

The involvement of animals in interventions and therapies for human clients is gaining increasing popularity but has also led to concern about the welfare of the integrated animals. However, there is no broadly accepted definition of welfare. In our opinion, the absence of stress in an animal is not a measure for welfare. Welfare is more visible as joy, or the eagerness to repeat behavior, a marker of motivation, the drive that humans pushes forward in their professional life. Until now, little attention has been paid to the motivation of the therapy animal.

Therefore, we make the following recommendations:

- Label the animal’s integration in interventions in terms of volunteer work or professional work and describe desired actions in a job description. This will help to qualify the competencies of the animal.
- Describe all primary and secondary benefits, such as renumeration, social plans, and retirement in the job description. This will ensure the long-term wellbeing of the therapy-assisting animal.
- Create a retirement plan but offer the animal to get involved in voluntary work such as presence and contact during daytime activities, walks during coaching sessions, grooming for children, presence during reading comprehension in school classes. These animals are well socialized, well raised and appreciate being with humans.
- Make the animal’s job, whether voluntary or professional, as enjoyable as possible. Mindfulness and play therapy are good examples.
- Stimulate neuroscientific research on the motivation of freely moving animals integrated in AAS, more specific on the mesolimbic reward pathway of dopamine. This will give more insight in the willingness of the animal to be or stay involved, and might reveal animals that are not motivated, but participate without resistance.
- Focus more research on the use of context-ethograms, animal ethograms related to the interaction with clients. This enables handlers to go beyond their intuition and score the animal’s behavior more objectively.

The question remains whether we can make interventions as attractive as possible for all parties, both the volunteers and the professional animals, the handler and the client. This requires insight and the recognition of positive interaction and positive emotions.

It is extremely difficult for handlers to distinguish between enthusiasm and stoic participation. Handlers need better tools to assess motivation. Translation of dopamine/motivation evoking interventions into context-ethograms may be of assistance when in doubt.

Future research on dopamine levels in animals in AAS, whether as volunteers or professionals, is needed to assess motivation. Based on these findings, context-ethograms can be developed for the diverse animals in order to better understand body language when dopamine is peaking.

## 9. Conclusions

Motivation is the driving force in successful cooperation between all dyads in AAS. The only one that is not easily readable is the animal involved, while he is the special added value to the intervention. Animal-friendly interventions can boost motivation, and more research on dopamine—the neurotransmitter involved in motivation and reward—is needed to reveal the difference between stoic cooperation and motivation. Motivation comes at different levels; a volunteering animal may be motivated to a different level or for a shorter time than a professional animal, which might go the extra mile.

Handlers and therapists need tools to assess the presence and absence of job-oriented-motivation and not just read pleasure in the animal. Context-ethograms can show the motivational body language of the animal in a certain context and should be made available for diverse handlers and therapists.
